# Propofol and Hypertriglyceridemia in the ICU: A Systematic Review and Meta-Analysis of Recent Observational Data

**DOI:** 10.7759/cureus.108132

**Published:** 2026-05-02

**Authors:** Andrew N Hendrix, Caroline H Kerrison, Zachary C Holley, Ankur Makani, Ashton Norris, Gunnar Orcutt, Logan B Carlyle, Christopher Watson, Phillip J Prest

**Affiliations:** 1 Department of Surgery, University of Virginia, Charlottesville, USA; 2 Department of Ophthalmology, University of Virginia, Charlottesville, USA; 3 Department of Surgery, Prisma Health-Midlands, University of South Carolina School of Medicine, Columbia, USA; 4 Department of Internal Medicine, Prisma Health-Midlands, Columbia, USA; 5 Department of Surgery, Prisma Health-Midlands, Columbia, USA; 6 Department of General Surgery, Prisma Health-Midlands, University of South Carolina School of Medicine, Columbia, USA

**Keywords:** hypertriglyceridemia, intensive care unit, length of stay, meta-analysis, propofol, sedation

## Abstract

While previous expert consensus guidelines by the American College of Critical Medicine recommend serum triglyceride monitoring after two consecutive days of propofol, the most recent Pain, Agitation/Sedation, Delirium, Immobility, and Sleep Disruption guidelines do not address monitoring triglyceride levels in patients on continuous propofol infusions. Recent cohort studies have shown that a majority of patients on continuous propofol lack triglyceride measurements. Our objective was to systematically summarize and compare the reported incidence of pancreatitis, hospital length of stay (LOS), intensive care unit (ICU) LOS, and hospital mortality in patients on continuous propofol infusions who developed hypertriglyceridemia (HTG) compared with those without HTG. Methods included Medical Subject Headings terms and keywords were used to construct a systematic search according to Preferred Reporting Items for Systematic reviews and Meta-Analyses (PRISMA) guidelines involving PubMed, Embase, Web of Science, Cochrane, and Cumulative Index to Nursing and Allied Health Literature (CINAHL). Inclusion criteria were papers published in English, since 2000, involving ICU patients ≥18 years old, who were on continuous propofol infusions. Six of the 790 studies identified (2,301 patients) were included. Pooled incidence of HTG was 28% with an average onset of 4.3 days. Both ICU LOS (pooled standardized mean difference (SMD) = 0.37, 95% CI: (0.16, 0.58), p < 0.0001, I² = 47%) and hospital LOS (pooled SMD = 0.21, 95% CI: (0.06, 0.35), p < 0.0001, I² = 0%) were increased in patients who developed HTG. Pooled incidence of pancreatitis was 1.3% with no significant difference between groups. In conclusion, HTG in ICU patients requiring continuous propofol infusions was associated with increased ICU and hospital LOS, with no significant differences in pancreatitis incidence or mortality. While this does not demonstrate causality due to its retrospective nature, it does point to an association of worse outcomes and hypertriglyceridemia in this setting.

## Introduction and background

Introduced in 1986, propofol is a widely used intravenous sedative agent, particularly favored for sedation in mechanically ventilated patients within the intensive care unit (ICU). Its rapid onset and favorable pharmacokinetic profile have established it as a first-line agent in this setting, as recommended by the 2025 Society of Critical Care Medicine Clinical Practice Guidelines for the Prevention and Management of Pain, Agitation/Sedation, Delirium, Immobility, and Sleep Disruption (PADIS) [1]. The exact mechanism of action of propofol is not entirely understood, but it is believed to exert its sedative effects through the positive modulation of the inhibitory neurotransmitter gamma-aminobutyric acid (GABA) via GABA-A receptors [2]. Additionally, there is evidence suggesting that propofol decreases glutamate release through N-methyl-D-aspartate (NMDA) inhibition [3]. Propofol is highly lipophilic, primarily consisting of linoleic acid, an omega-6 long-chain polyunsaturated fatty acid, formulated in a 10% oil-in-water lipid emulsion [3]. While largely safe, this formulation is associated with certain risks, most notably dose-dependent hypotension, which is particularly pronounced in volume-depleted patients [3]. More infrequent complications include hypertriglyceridemia (HTG), pancreatitis, and propofol infusion syndrome--a serious condition characterized by metabolic acidosis, rhabdomyolysis, hyperkalemia, hepatomegaly, renal failure, hyperlipidemia, arrhythmias, and rapidly progressive cardiac failure [4].

The pathophysiological link between propofol-induced HTG and the development of acute pancreatitis involves the hydrolysis of triglycerides by pancreatic lipase, excessive formation of free fatty acids leading to inflammatory changes and capillary injury, and the roles of hyperviscosity and ischemia [5]. Although the incidence is not well defined, studies have examined the relationship between HTG and acute pancreatitis in patients receiving propofol. They highlight potential risk factors, including cumulative propofol dose, duration of administration, younger age, higher body mass index (BMI), increased severity of illness, and concomitant medications [6]. Despite the recognized risks, there remains a lack of comprehensive guidelines and higher-quality studies surrounding the management of propofol-induced HTG in ICU patients. While Critical Care Medicine’s clinical practice guidelines in 2013 recommended serum triglyceride monitoring after two consecutive days of propofol [7], the most recent PADIS guidelines in 2025 do not address the monitoring of triglyceride levels in patients on continuous infusion of propofol [1]. This discrepancy in clinical guidelines is underscored by recent cohort studies that revealed 73-84% of patients on continuous propofol infusions do not undergo routine triglyceride monitoring, potentially compromising patient outcomes [8]. Addressing this issue is crucial, as patients with elevated triglyceride levels are potentially at risk for worse outcomes, including higher rates of pancreatitis, longer ICU and hospital stays, increased ventilator days, and higher mortality rates. This systematic review and meta-analysis aims to investigate the association of propofol-induced HTG with patient outcomes, including pancreatitis incidence, hospital length of stay (LOS), ICU LOS, and hospital mortality, with the goal of informing future clinical guidelines and improving care for critically ill patients. Given that most of the existing literature is retrospective and observational data, there is a need for higher-quality evidence. The authors are hopeful that this review of existing literature reveals any inconsistencies in the literature and illuminates potential shortcomings for future studies. 

## Review

Methods 

Literature Search

Guidelines from the Preferred Reporting Items for Systematic review and Meta-Analyses (PRISMA) [9] were used to conduct a systematic review of original research studies reporting the use of continuous propofol in ICU patients, which was performed on September 30, 2024. The protocol for this review was registered prior to data collection on the International Prospective Register of Systematic Reviews (PROSPERO) (Registration ID. CRD420251029663). Medical Subject Headings (MeSH) terms and keywords were used to construct a systematic search involving the following databases: PubMed, Embase, Web of Science, Cochrane, and Cumulative Index to Nursing and Allied Health Literature (CINAHL) (Table 1). This systematic review was based solely on publicly available data and, accordingly, was deemed exempt from review by our institutional review board.

**Table 1:** Database search strategies.

Database search strategy	
Pubmed/Medline	Search: ((intensive care unit OR critical care OR resuscitation OR mechanical ventilation OR pressors OR ( "Intensive Care Units/statistics and numerical data"[Mesh] OR "Intensive Care Units/supply and distribution"[Mesh] OR "Intensive Care Units/trends"[Mesh] )) AND (Propofol OR sedative OR anesthetic OR comatose OR intubated OR ( "Propofol/adverse effects"[Mesh] OR "Propofol/metabolism"[Mesh] OR "Propofol/pharmacokinetics"[Mesh] OR "Propofol/standards"[Mesh] OR "Propofol/therapeutic use"[Mesh] OR "Propofol/toxicity"[Mesh] ))) AND (Hypertriglyceridemia OR hyperlipidemia OR hyperchylomicronemia OR hyperlipoproteinemia OR pancreatitis OR ( "Hypertriglyceridemia/chemically induced"[Mesh] OR "Hypertriglyceridemia/complications"[Mesh] OR "Hypertriglyceridemia/drug therapy"[Mesh] OR "Hypertriglyceridemia/epidemiology"[Mesh] OR "Hypertriglyceridemia/etiology"[Mesh] OR "Hypertriglyceridemia/mortality"[Mesh] OR "Hypertriglyceridemia/physiopathology"[Mesh] )) Filters: Adult: 19+ years, from 2004 - 2024
Web of Science	((ALL=(intensive care unit OR critical care OR resuscitation OR mechanical ventilation OR pressors)) AND ALL=(Propofol OR sedative OR anesthetic OR comatose OR intubated)) AND ALL=(Hypertriglyceridemia OR hyperlipidemia OR hyperchylomicronemia OR hyperlipoproteinemia OR pancreatitis ) and 2024 or 2023 or 2022 or 2021 or 2020 or 2019 or 2018 or 2017 or 2016 or 2015 or 2014 or 2013 or 2012 or 2011 or 2010 or 2009 or 2008 or 2007 or 2006 or 2005 or 2004 (Publication Years)
Cochrane	((ALL=(intensive care unit OR critical care OR resuscitation OR mechanical ventilation OR pressors)) AND ALL=(Propofol OR sedative OR anesthetic OR comatose OR intubated)) AND ALL=(Hypertriglyceridemia OR hyperlipidemia OR hyperchylomicronemia OR hyperlipoproteinemia OR pancreatitis ) and 2024 or 2023 or 2022 or 2021 or 2020 or 2019 or 2018 or 2017 or 2016 or 2015 or 2014 or 2013 or 2012 or 2011 or 2010 or 2009 or 2008 or 2007 or 2006 or 2005 or 2004 (Publication Years)
CINAHL	((ALL=(intensive care unit OR critical care OR resuscitation OR mechanical ventilation OR pressors)) AND ALL=(Propofol OR sedative OR anesthetic OR comatose OR intubated)) AND ALL=(Hypertriglyceridemia OR hyperlipidemia OR hyperchylomicronemia OR hyperlipoproteinemia OR pancreatitis ) and 2024 or 2023 or 2022 or 2021 or 2020 or 2019 or 2018 or 2017 or 2016 or 2015 or 2014 or 2013 or 2012 or 2011 or 2010 or 2009 or 2008 or 2007 or 2006 or 2005 or 2004 (Publication Years)
Embase	((ALL=(intensive care unit OR critical care OR resuscitation OR mechanical ventilation OR pressors)) AND ALL=(Propofol OR sedative OR anesthetic OR comatose OR intubated)) AND ALL=(Hypertriglyceridemia OR hyperlipidemia OR hyperchylomicronemia OR hyperlipoproteinemia OR pancreatitis ) and 2024 or 2023 or 2022 or 2021 or 2020 or 2019 or 2018 or 2017 or 2016 or 2015 or 2014 or 2013 or 2012 or 2011 or 2010 or 2009 or 2008 or 2007 or 2006 or 2005 or 2004 (Publication Years

Inclusion and Exclusion Criteria

Eligible articles included peer-reviewed studies published in English after the year 2000, encompassing observational, experimental, and randomized controlled trials that involved ICU patients aged 18 years or older receiving continuous propofol infusions and reported triglyceride levels. While the ATP III guidelines from the National Cholesterol Education Program classify triglyceride levels above 500 mg/dL as severely elevated [10], this study employed a more conservative definition. Informed by the earlier 1993 ATP II recommendations [11], we defined hypertriglyceridemia as a serum triglyceride concentration of ≥400 mg/dL. Case reports, abstracts, systematic reviews, and non-English articles were excluded.

Data Extraction and Quality Assessment

Based on the established inclusion and exclusion criteria, two authors (CK and AH) independently reviewed the titles, abstracts, and available full-text articles to determine which studies would be included. In cases of disagreement due to differing interpretations, a third investigator (LC) was consulted to mediate and facilitate consensus through discussion. Inter-rater agreement was assessed by % agreement. The following information was extracted from all of the trials using a standardized Microsoft Excel form: first author, year of publication, study design, sample size, definition for continuous propofol infusion, diagnostic cut-off for HTG, and patient characteristics. Patient characteristics include age, sex at birth, BMI, and Acute Physiology and Chronic Health Evaluation (APACHE) score [12]. Primary endpoints for the study included hospital LOS, ICU LOS, hospital mortality, and pancreatitis incidence. If a study did not report one or more of the predefined primary outcomes, that study was excluded from the meta-analysis for that specific outcome but still included in the analysis of any other outcomes it reported. This approach ensured that all available data were utilized without introducing bias through imputation or selective inclusion.

Two authors (CK and AH) independently assessed the risk of bias in accordance with the Risk Of Bias In Non-randomized Studies of Interventions (ROBINS-I) tool [13]. We chose ROBINS-I over alternatives like Grading, Review, Assess, and Develop (GRADE) or the Newcastle-Ottawa tool because, although our protocol initially allowed for randomized trials, the available evidence was entirely observational, making ROBINS-I’s framework ideally suited to capture the inherent methodological challenges in these studies [13]. The risk of bias in each item was graded as “low”, “moderate”, “serious”, or “critical”.

Statistical Analysis

Statistical meta-analysis was conducted using the meta package v4.17-0 in R: A language and environment for statistical computing version 4.2.1. R Foundation for Statistical Computing, Vienna, Austria (R open-source software) [14]. Standardized mean differences (SMDs) were calculated for continuous outcomes using the difference in group means divided by a pooled estimate of standard deviation (SD). Because most included studies did not report SDs, we used a conservative estimation approach that assumed a consistent pooled SD across groups, following guidance from the Cochrane Handbook for Systematic Reviews of Interventions [15]. For dichotomous outcomes, odds ratios (ORs) were calculated from reported event rates in HTG and non-HTG groups. Log-transformed ORs and their variances were then used for meta-analysis. Forest plots were utilized to visually summarize these data. Random effects models were used to calculate pooled effect sizes and account for potential heterogeneity between studies. All pooled estimates were reported with corresponding 95% confidence intervals (CIs). Heterogeneity was quantified using the I² statistic with I² values > 50% indicating statistically significant heterogeneity. No subgroup analyses were performed to identify sources of heterogeneity. 

Results 

Literature Identification and Search Results

**Figure 1:**
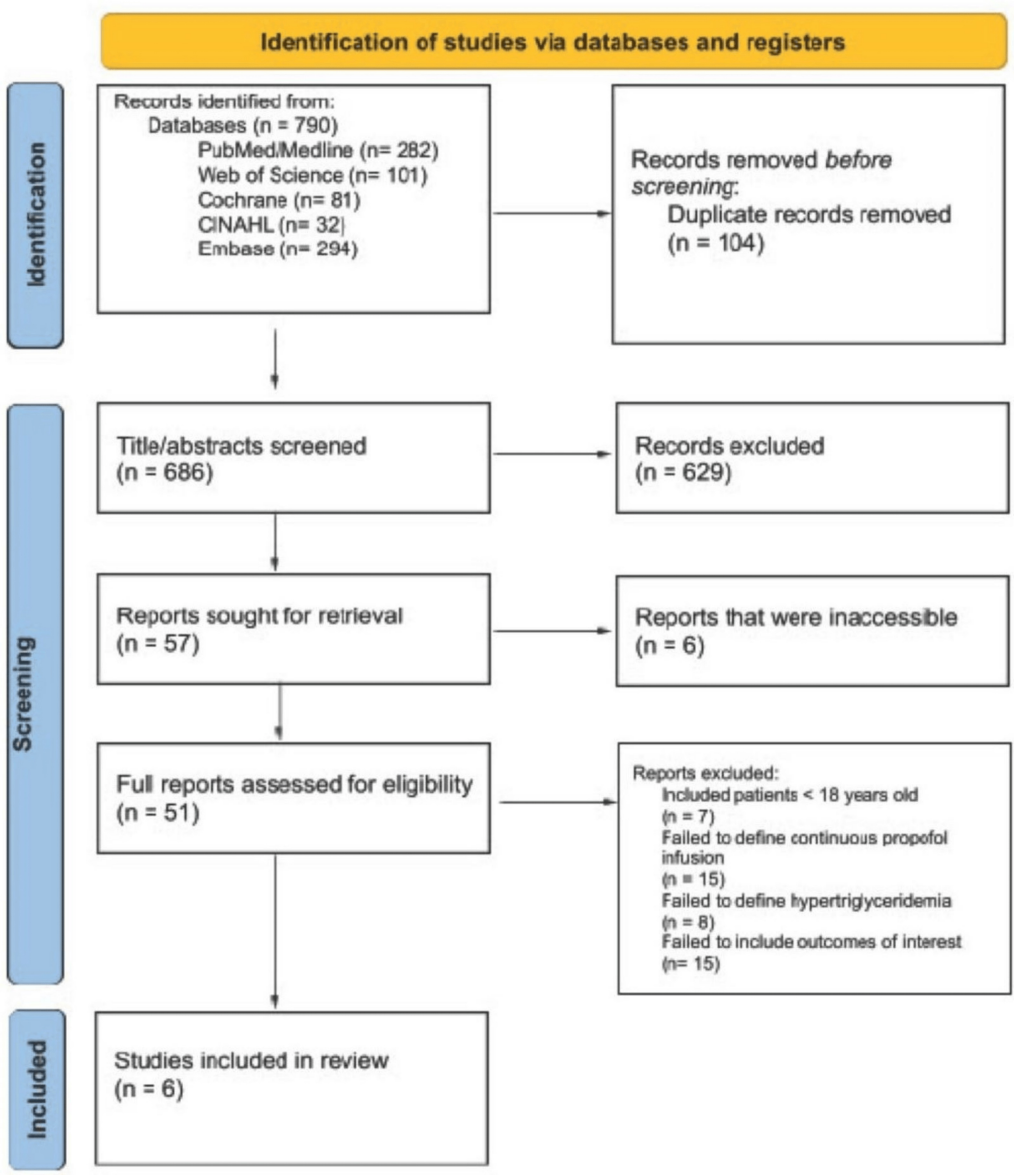
PRISMA flow chart. PRISMA: Preferred Reporting Items for Systematic reviews and Meta-Analyses.

Based on our comprehensive search strategy using multiple databases, a total of 790 records were initially identified from PubMed/Medline (n = 282), Web of Science (n = 101), Cochrane (n = 81), CINAHL (n = 32), and Embase (n = 294). After removing 104 duplicate records, 686 unique records were available for screening. In the initial title and abstract screening, which achieved a 92% inter-reviewer agreement, 629 records were excluded because they did not meet the inclusion criteria. During the full-text review, which achieved an 87% inter-reviewer agreement, 57 articles were assessed for eligibility. Articles were excluded at this stage for the following reasons: unable to access full text (n = 6), inclusion of patients under 18 years of age (n = 7), failure to define continuous propofol infusion (n = 15), failure to define HTG (n = 8), and failure to include the outcomes of interest (n = 15). Ultimately, six studies met the final criteria for inclusion [8,16-20]. Figure 1 shows the workflow for the selection of studies according to the PRISMA guidelines [9].

Study Characteristics

The six selected studies describe a total of 2,301 ICU patients who were on continuous propofol infusions between January 2003 and September 2021. All studies employed a retrospective design with sample sizes ranging from 106 to 1,365 participants. Definitions for continuous propofol infusion varied among the studies; three studies [16-18] defined it as infusions lasting more than 12 to 24 hours, one study used a threshold of more than four hours [20], one study [19] used a threshold of more than 48 hours, and one study [8] included any duration of administration. HTG was most commonly defined as serum triglyceride levels exceeding 400 mg/dL, although one study [16] applied a higher threshold of more than 500 mg/dL. The mean incidence of propofol-induced HTG across all studies was 28% (SD: 9.8%), and the average onset was 4.3 days (SD: 0.91 days). It should be noted that this pooled incidence estimate should be interpreted cautiously, given heterogeneous thresholds for hypertriglyceridemia and differences in monitoring practices. Study characteristics are further described in Table 2. All articles were of at least moderate quality, as assessed using the ROBINS-I tool by Cochrane Methods [13], as shown in Table 3.

**Table 2:** Study characteristics. ICU: intensive care unit; HTG: hypertriglyceridemia.

Author	Date of data collection	Study design	Setting	Sample size	Continuous propofol definition (hours)	HTG definition (mg/dl)	HTG incidence (%)	HTG onset (days)
Stallworth et al. (2023) [16]	July 2013-September 2021	Retrospective cohort	Medical ICU	167	>24	>500	34.7	4.8
Witenko et al. (2022) [17]	March 2020-April 2020	Retrospective cohort	Did not specify ICU type	252	>12	>400	38.9	3.8
Devlin et al. (2005) [18]	January, 2003-March 2004	Retrospective cohort	Medical or surgical ICU	159	>24	>400	18	2.25
Rubino et al. (2023) [19]	July 2019-July 2022	Retrospective cohort	Medical or surgical ICU	252	>48	>400	16.3	-
Kovacevic et al. (2021) [20]	March, 2020-April 2020	Retrospective cohort	Did not specify ICU type	106	>4	>400	56.7	1.9
Pancholi et al. (2023) [8]	January 2016-April 2021	Retrospective cohort	Did not specify ICU type	1,365	Any duration	>400	20.8	4.77
Total				2,301			28.0	3.50

**Table 3:** ROBINS-I (risk of bias in non-randomized studies-of interventions).

Author	Bias in the classification of interventions	Bias in the selection of participants into the study (or into the analysis)	Bias due to deviations from intended interventions	Bias due to missing data	Bias in the measurement of the outcome	Bias in the selection of the reported result
Stallworth et al. (2023) [16]	Low	Low	Low	Low	Low	Low
Witenko et al. (2022) [17]	Low	Moderate	Low	Low	Low	Low
Devlin et al. (2005) [18]	Low	Low	Low	Moderate	Low	Low
Rubino et al. (2024) [19]	Low	Low	Low	Serious	Low	Low
Kovacevic et al. (2021) [20]	Low	Moderate	Low	Low	Low	Low
Pancholi et al. (2023) [8]	Low	Serious	Low	Moderate	Low	Low

Patient Characteristics

Of the 2,301 patients, the pooled mean age was 50.1 years (SD: 5.4 years), and 59.4% were male (SD: 5.2%). BMI was reported in three studies [8,16,20], with a pooled mean BMI of roughly 30.5 kg/m² (SD: ~1.3 kg/m²). Four studies [8,17,18,20] also provided APACHE II scores to gauge illness severity at ICU admission. In three of these studies [17,18,20], the reported mean APACHE II scores ranged from 18.0 to 27.0; however, Pancholi et al. reported markedly elevated APACHE II scores of 70.4 in the HTG group and 66.3 in the non-HTG group. Patient characteristics are further described in Table 4.

**Table 4:** Patient characteristics. HTG: hypertriglyceridemia; BMI: body mass index; APACHE II: Acute Physiology and Chronic Health Evaluation II.

Author	Age (years)			BMI (kg/m^2^)			Female (%)			APACHE II score	
	Total	HTG	Non-HTG	Total	HTG	Non-HTG	Total	HTG	Non-HTG	HTG|	Non-HTG
Stallworth et al. (2023) [16]	45	45	46	33.7	33.9	33.6	41.3	32.80	45.9	-	-
Witenko et al. (2022) [17]	67	-	-	-	-	-	28	-	-	26	27
Devlin et al. (2005) [18]	-	53	41	-	-	-	-	48	43	19	17
Rubino et al. (2023) [19]	-	-	-	-	-	-	-	-	-	-	-
Kovacevic et al. (2021) [20]	66	60	70	27.8	28	27.8	36.8	35	39.9	21	21
Pancholi et al. (2023) [8]	61.5	55.5	61.5	30	33.4	30.5	43.2	37	42.4	70.4	66.3

ICU Length of Stay (LOS)

**Figure 2:**
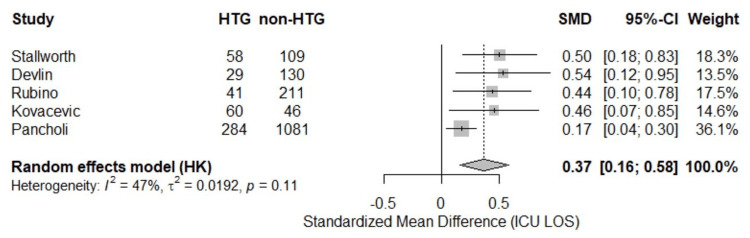
Intensive care unit length of stay (forest plot). HTG: hypertriglyceridemia; ICU: intensive care unit; LOS: length of stay; SMD: standardized mean difference; CI: confidence interval; Stallworth et al. (2023) [16]; Devlin et al. (2005) [18]; Rubino et al. (2023) [19]; Kovacevic et al. (2021) [20]; Pancholi et al. (2023) [8].

Table 5 reports primary outcomes across included studies. Average ICU LOS for HTG patients was 17.3 days compared to 12.3 days for non-HTG patients. A random-effects meta-analysis of the five studies [8,16,18,19,20] reporting ICU LOS demonstrated a statistically significant increase in ICU LOS among patients who developed HTG while on continuous propofol infusion compared to those who did not (pooled SMD = 0.37; 95% CI: 0.16 to 0.58; p < 0.0001) (Figure 2). Moderate heterogeneity was observed across studies (I² = 47.0%), although the test for heterogeneity was not statistically significant (p = 0.11). 

Hospital LOS

**Figure 3:**
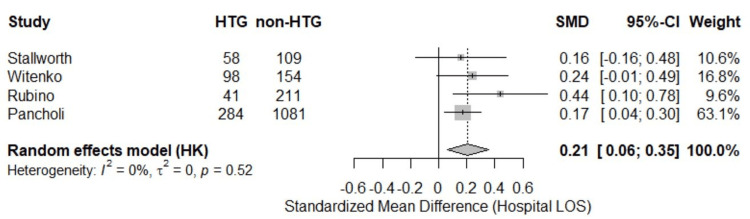
Hospital length of stay (forest plot). HTG: hypertriglyceridemia; LOS: length of stay; SMD: standardized mean difference; CI: confidence interval; Stallworth et al. (2023) [16]; Witenko et al. (2022) [17]; Rubino et al. (2023) [19]; Pancholi et al. (2023) [8].

Average hospital LOS was 35.0 days for patients who developed HTG versus 27.8 days for non-HTG patients. A random-effects meta-analysis of the four studies [8,16,17,19] evaluating hospital LOS found that patients who developed HTG while receiving continuous propofol infusion had a significantly longer hospital LOS compared to those who did not (pooled SMD = 0.21; 95% CI: 0.06 to 0.35; p < 0.0001) (Figure 3). No heterogeneity was detected across studies (I² = 0.0%), and the test for heterogeneity was not statistically significant (p = 0.52). 

Hospital Mortality

**Figure 4:**
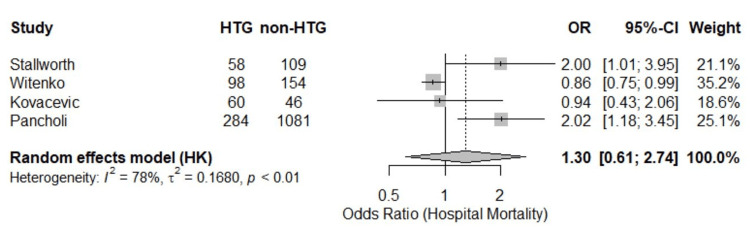
Hospital mortality (forest plot). HTG: hypertriglyceridemia; OR: odds ratio; CI: confidence interval; Stallworth et al. (2023) [16]; Witenko et al. (2022) [17]; Kovacevic et al. (2021) [20]; Pancholi et al. (2023) [8].

The average mortality rate for HTG patients was 42.6%, compared to 35.3% for non-HTG patients. A random-effects meta-analysis of the four studies [8,16,17,20] evaluating hospital mortality in patients receiving continuous propofol infusion showed no statistically significant difference in mortality between those who developed HTG and those who did not (pooled odds ratio = 1.30; 95% CI: 0.61 to 2.74; p = 0.29) (Figure 4). Substantial heterogeneity was observed across studies (I² = 78%), and the test for heterogeneity was statistically significant (p = 0.003).

Pancreatitis Incidence

The pooled incidence of pancreatitis among patients on propofol was very low, 1.3%, with no obvious significant difference between the HTG and non-HTG groups. Unfortunately, data limitations prevented a reliable meta-analysis, and drawing conclusions about pancreatitis in this particular paper should be used with caution.

**Table 5:** Primary outcomes across studies. ICU: intensive care unit; LOS: length of stay; HTG: hypertriglyceridemia.

Author	ICU LOS HTG	ICU LOS non-HTG	p- value	Hospital LOS HTG	Hospital LOS non-HTG	p- value	Hospital mortality HTG	Hospital mortality non-HTG	p-value	Pancreatitis incidence HTG	Pancreatitis incidence non-HTG
Stallworth et al. (2023) [16]	26.5	23	0.002	32.5	30	0.34	51.7	34.9	0.047	0	0.9
Witenko et al. (2022) [17]	-		-	42.5	35	0.063	30.6	33.8	0.042	0	0
Devlin et al. (2005) [18]	8.6	4.1	0.01	-	-	-	-	-	-	10.3	-
Rubino et al. (2023) [19]	18	12	0.01	29	21	0.01	-	-	-	0	0
Kovacevic et al. (2021) [20]	17.5	12.9	0.0198	-	-	-	33.3	34.7	0.8759	1.7	-
Pancholi et al. (2023) [8]	15.8	9.4	0.01	35.9	25.1	0.01	54.9	37.6	0.01	3.3	1.2
Total	17.28	12.28	<0.001	34.975	27.775	<0.001	42.625	35.25	0.1888	2.55	0.525

Discussion

Key Findings

This is the first systematic review and meta-analysis to evaluate outcomes in ICU patients on continuous propofol infusions who develop HTG. Our findings demonstrated that the development of HTG in ICU patients receiving continuous propofol infusions was associated with a modest and statistically significant increase in both ICU and hospital length of stay, suggesting that elevated triglyceride levels are associated with prolonged recovery times. This could possibly reflect HTG as a marker of increased illness severity and not a direct causal agent. In contrast, no significant association was observed between HTG and hospital mortality. Additionally, there did not appear to be a relationship between HTG and pancreatitis. While the data highlight the rarity of pancreatitis in this patient population, due to the low incidence and lack of data continuity, definitive conclusions were not able to be drawn. The heterogeneity of effects was minimal for hospital LOS, moderate but not significant for ICU LOS, and substantial for hospital mortality.

Relationship to Previous Studies

Our findings are broadly supported by a recent multicenter cohort study by Heybati et al. [21], which reported a 21.7% incidence of HTG among ICU patients receiving continuous propofol, with an average onset of 4.5 days. This study also found that HTG was associated with increased ICU and hospital LOS irrespective of pancreatitis development. Additionally, Heybati et al. reported a similar pancreatitis incidence of 1.2%; however, they found higher rates among HTG patients compared to non-HTG patients (3.2% vs. 0.7%, p < 0.001). This study was not included in our quantitative synthesis because it was published well after the end of our literature search window.

Implications

The findings from this meta-analysis emphasize the significant impact of propofol-induced HTG on ICU and hospital LOS, even though pancreatitis as a complication appeared to be rare. This distinction is crucial; while the fear of pancreatitis has historically driven triglyceride monitoring, our results suggest that this relationship may not be clinically relevant. This study was also not powered to draw conclusions regarding pancreatitis. However, given that HTG is associated with significantly longer ICU and hospital stays, triglyceride levels may provide a practical marker for clinicians to consider alternative sedation strategies. While this paper does not specifically review interventional evidence supporting various sedation agents, switching to agents such as dexmedetomidine at the early onset of HTG might reduce exposure to propofol's lipid content [1]. The practice of spontaneous awakening trials and dose minimization should also be considered as part of a comprehensive approach to manage triglyceride levels, especially in those with a predisposition to rapid triglyceride elevation [6]. The proactive monitoring and management of HTG are thus not about mitigating pancreatitis but are instead focused on optimizing patient outcomes through better prediction and adjustment of treatment strategies. While Critical Care Medicine’s clinical practice guidelines in 2013 recommended serum triglyceride monitoring after 2 consecutive days of propofol [7], the most recent PADIS guidelines in 2025 fail to address monitoring of triglyceride levels in patients on continuous infusion of propofol [1]. The evidence presented in this study highlights a need for prospective studies to better inform updated recommendations on the topic.

Limitations

Despite the insights provided by this review, several limitations must be acknowledged. First, despite a comprehensive database search, only a few studies were eligible for inclusion. Only including published (i.e., peer-reviewed) studies may introduce some publication bias. Additionally, the included studies consisted entirely of retrospective cohort studies. The potential biases in retrospective analyses may affect the generalizability of our findings. Due to the nature of retrospective studies, there is a risk of missing data due to selective reporting, in addition to the inability to establish temporality between HTG and the observed outcomes. Specifically, it remains uncertain whether HTG is a cause or consequence of a more complicated ICU course. Patients who develop HTG may simply be sicker at baseline, with a higher burden of illness that predisposes them both to elevated triglyceride levels and to longer ICU and hospital stays. In this case, HTG could be seen more as a marker of severe illness rather than an active contributor to adverse outcomes. This interpretation aligns with observational biases inherent in retrospective analyses, where sicker patients are often subject to more extensive interventions, including higher doses or longer durations of propofol, which could confound the association between HTG and prolonged LOS. The absence of subgroup or sensitivity analyses limits interpretation, particularly in the presence of significant clinical and methodological heterogeneity. On the other hand, there is also a plausible biological mechanism by which HTG actively contributes to prolonged recovery. Elevated triglyceride levels are associated with increased blood viscosity, capillary stasis, and subsequent tissue hypoperfusion, which could impair overall recovery and increase the need for prolonged critical care [5,22,23]. This mechanism suggests that HTG itself might exacerbate the clinical situation, leading to longer ICU and hospital stays. Differentiating these potential explanations is challenging given the retrospective nature of the included studies. The use of the HTG threshold of ≥400 mg/dL instead of the more contemporary ≥500 mg/dL may affect incidence estimates and comparability across studies. We also recognize that restriction to English-language publications introduces potential language and publication bias.

## Conclusions

In this systematic review and meta-analysis, we found that roughly one in four patients on continuous propofol infusion will develop propofol-induced HTG within the first couple of days of infusion. Development of HTG in ICU patients receiving continuous propofol infusions was associated with longer ICU and hospital length of stay, with no significant difference observed in pancreatitis incidence or hospital mortality. Due to the limitations described above regarding the nature of retrospective data in this review, interpretation of results should be used with caution. Further high-quality studies are needed to elucidate causality. Large, multicenter observational studies are currently ongoing.
